# The Efficacy of β-Carotene in Cow Reproduction: A Review

**DOI:** 10.3390/ani14142133

**Published:** 2024-07-22

**Authors:** Hiroki Mitsuishi, Masato Yayota

**Affiliations:** 1 The United Graduate School of Agricultural Science, Gifu University, Gifu 501-1193, Japan; mitsuishi.hiroki.p7@s.gifu-u.ac.jp; 2 Faculty of Applied Biological Sciences, Gifu University, Gifu 501-1193, Japan; 3 Education and Research Center for Food Animal Health, Gifu University (GeFAH), Gifu 501-1193, Japan

**Keywords:** β-carotene, cattle, reproduction

## Abstract

**Simple Summary:**

β-Carotene is an abundant carotenoid in plants that functions as an antioxidant and a primary precursor of vitamin A in cattle. In addition to its role as a vitamin A precursor, β-carotene affects reproduction. β-carotene supports reproductive function, especially luteal function, in cattle by protecting synthetic enzymes related to sex steroid hormone production from reactive oxygen species through its antioxidant properties. β-carotene supplementation supports the ovarian cycle in cows by suppressing oxidative stress and enhancing immune function. Future research and field applications should consider β-carotene sufficiency and the balance between β-carotene intake from roughage and β-carotene consumption in the body and possible factors affecting the effectiveness of β-carotene in cattle reproduction.

**Abstract:**

β-carotene supplementation improves the reproductive performance of cattle. However, the research results on this topic have been inconsistent, and no clear conclusion has been reached. In previous reviews of this topic, the functional mechanism of β-carotene in reproduction remained unclear, but subsequent studies have shown that the antioxidant effects of β-carotene protect enzymes involved in ovarian sex steroid hormone production from the effects of oxygen radicals. This role consequently affects normal ovarian follicle dynamics, maintenance of luteal function, and the estrous cycle, and indirectly improves reproductive performance by preventing perinatal diseases and facilitating recovery from these diseases. Several factors must be considered in feeding management to determine whether β-carotene supplementation is effective for improving reproductive performance in cows. The same is true when the animal consumes a large amount of the antioxidant β-carotene due to lactation, aging, or season. Therefore, it is important to consider the balance between the supply and consumption of β-carotene and evaluate whether β-carotene supplementation has an effect on reproductive performance in cows.

## 1. Introduction

β-carotene is an abundant carotenoid in plants that functions as an antioxidant and a primary precursor of vitamin A in cattle. In addition to its role as a vitamin A precursor, β-carotene has been studied as a nutrient that may affect reproduction in cattle for years [1]. However, according to the NASEM [1], the effects of β-carotene supplementation on reproduction in dairy cows are highly inconsistent. Some studies have suggested that β-carotene supplementation is effective for improving reproductive function or performance, while other studies have denied its effectiveness. This inconsistency may be caused by differences in the basal β-carotene intake and vitamin A status of the cows. Thus, the nutrient requirements for β-carotene in dairy cattle have not been determined to date. Moreover, there is less information about the function of β-carotene in beef cattle than in dairy cattle, especially concerning reproduction. Accordingly, even in the latest version of the guideline, the nutrient requirements of beef cattle [2] have not been considered.

The effect of β-carotene on cattle reproduction was reviewed in the 1980s by Hemken and Bremel (1982) [3] and Hurley and Doane (1989) [4]. Since then, many researchers have attempted to determine the function and mechanism of β-carotene in cattle reproduction. More recently, Shastak and Pelletier (2024) [5] reported that β-carotene had effects on immunity, antioxidant capacity, and fertility in poultry, swine, and cattle. They also noted inconsistent results regarding the effects of β-carotene supplementation on reproduction and that further research is needed to determine the underlying mechanisms involved.

This review aims to (1) summarize the historical and recent progress in identifying novel mechanisms of β-carotene actions in cattle reproduction and (2) discuss the factors that lead to inconsistencies in the effects of β-carotene supplementation on cattle reproduction in terms of physiological mechanisms and feeding management.

## 2. Recent Progress in β-Carotene Research on Cattle Reproduction

**Figure 1:**
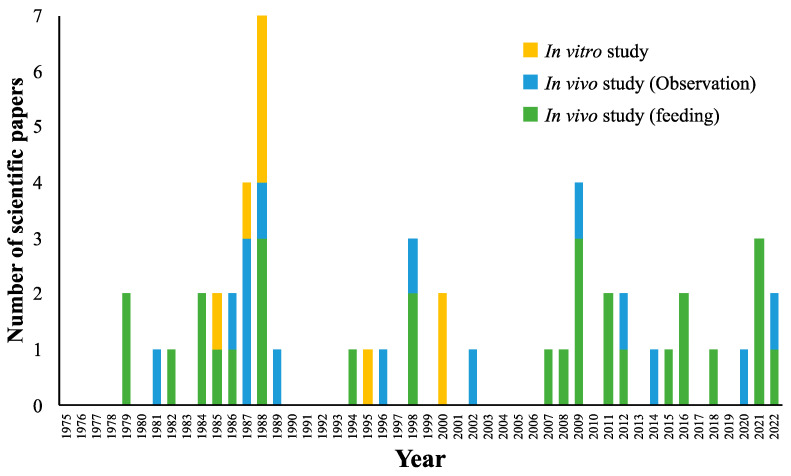
Annual number of scientific papers on β-carotene and cattle reproduction. English language articles were obtained from Google Scholar (http://scholar.google.com/), ScienceDirect (https://www.sciencedirect.com), and PubMed (https://pubmed.ncbi.nlm.nih.gov) using the following keywords: β-carotene, reproduction, and cow.

We identified 52 studies describing research methods, such as in vitro, observational, and feeding studies, published since 1975 (52 papers) in international journals, and they are summarized in Figure 1. These studies include English language articles identified in Google Scholar (http://scholar.google.com/), ScienceDirect (https://www.sciencedirect.com), and PubMed (https://pubmed.ncbi.nlm.nih.gov) using the following keywords: β-carotene, reproduction, and cow.

Feeding and observational studies involving β-carotene supplementation were conducted consistently from the 1980s to the present, and this has been a topic of tremendous interest in recent years. Additionally, in vitro cell culture studies have been conducted from approximately 1985 to 2000 to determine the mechanism of action of β-carotene, especially its antioxidant effects, in ovarian steroidogenesis. This research trend suggests that the function of β-carotene in cattle reproduction has garnered the attention of researchers for many years. However, this finding also implies that the function and mechanism of this process remain to be explained.

## 3. Brief History of Early Research on the Effects of β-Carotene Supplementation on Cattle Reproduction

β-carotene and vitamin A have long been studied as nutrients for cattle reproduction. Early studies were conducted mainly in dairy cattle. According to the review by Hemken and Bremel (1982) [3], the literature published before 1950 was focused primarily on the function of total carotene rather than that of β-carotene in cattle reproduction. Research concerning this topic shifted from carotenes to vitamin A in the following two decades. Research on the efficacy of β-carotene for cattle reproduction began in the 1970s–1980s [3]. This may be because the synthesis of retinyl esters became possible, and they are commonly used as a vitamin A supplement for cattle. In a paper summarizing several German studies, Lotthammer (1979) [6] suggested the importance of the antioxidant effect of β-carotene. Researchers then sought to determine not only the role of β-carotene as a vitamin A precursor but also whether β-carotene itself has a pronounced effect on cattle reproduction. Some researchers have reported improvements in cattle reproductive function or performance, such as clear estrus intensity [7], a short interval between estrus and ovulation [6], a high conception rate [6,8], a decrease in the number of insemination events per conception [6,9] and calving interval [9], a low incidence of follicular cysts [6], and a low early mortality rate [6] caused by or associated with β-carotene feeding. In contrast, other researchers did not find any positive effect of β-carotene on cattle reproduction [10,11,12,13,14,15]. Some reasons for this discrepancy are the various experimental conditions, including differences in the age of the study animals, the stage of lactation when β-carotene supplementation started, the length of the β-carotene supplementation, the form of vitamin A supplementation, and the β-carotene content in the basal diet [4]. Considering the findings of earlier studies, Hemken and Bremel (1982) [3] and Hurley and Doane (1989) [4] suggested that elucidating the detailed physiological mechanisms of β-carotene function in cattle reproduction is the key to solving this problem.

## 4. Mechanism of Action of β-Carotene in the Reproductive Function of Cattle at the Cellular Level

In vitro and culture studies of ovarian tissue were conducted around the 1990s to determine the physiological association between β-carotene and reproduction. In vitro studies of ovarian tissue are effective in clarifying the role of β-carotene in the ovary, which is the center of reproductive function, because this approach is not affected by external factors, such as feeding and the reproductive method; environmental factors, such as oxidative stress due to heat stress; or internal factors, such as the activity of other organs or the general condition of the body, the effects of which cannot be excluded when performing in vivo studies.

An earlier study by Pethes et al. (1985) [16] reported that 500 mg/day of synthetic β-carotene supplementation to cows for two months increased the blood β-carotene concentration. The cows were administered PG twice for estrus synchronization, and then corpus luteum was collected during the luteal phase when slaughtering. They also found that in vitro progesterone (P_4_) production was increased in luteal cells after stimulation with human chorionic gonadotropin (hCG). A similar study also confirmed that P_4_ synthesis was enhanced in luteal cells obtained from cows fed β-carotene [17].

In observational studies, in vitro P_4_ production in bovine luteal cells stimulated by luteinizing hormone (LH) significantly correlated with blood β-carotene concentration in Holstein cows [18]. A more detailed cell culture study [19] indicated that β-carotene in bovine corpus luteum cells prevented the inactivation of side-chain cleavage enzymes (P450scc), which was associated with steroid synthesis by covalent nondisulfide cross-linking with the electron donor adrenodoxin. This means that β-carotene protects cholesterol P450scc from oxygen-free radical damage. Rodgers et al. (1995) [20] also reported that β-carotene in culture medium promoted basal and cholesterol-stimulated P_4_ secretion by bovine corpus luteum cells. However, excess β-carotene in a culture medium rapidly suppressed P_4_ production, possibly due to the luteolytic effect of a high concentration of β-carotene [21,22]. Furthermore, in vitro P_4_ synthesis is enhanced by β-carotene, even in granulosa cells [23]. The relevance of β-carotene in vitro antioxidant action and sex steroid hormone synthesis proposed by these studies is still a leading hypothesis for the basal physiological mechanism of β-carotene in cow reproduction.

The involvement of β-carotene in P_4_ production revealed by in vitro studies was confirmed by observational studies at the level of individual cattle. The concentration of β-carotene in the corpus luteum, which increases in the luteal phase and rapidly decreases in the luteolytic phase with changes in antioxidant activity in cows, is positively correlated with the cyclic change in P_4_ in plasma and the cytochrome P450scc enzyme in the corpus luteum [24]. The plasma β-carotene concentration was significantly and strongly correlated with that in the follicular fluid (r = 0.836) and corpus luteum (r = 0.818), and the β-carotene concentration in the corpus luteum was positively correlated with indices of luteal function, including plasma P_4_ concentration (r = 0.339), luteal diameter (r = 0.428), and luteal weight (r = 0.423). In contrast, plasma and corpus luteum vitamin A were not associated with luteal function [25]. Therefore, Haliloglu et al. (2002) [25] suggested that β-carotene in the plasma and corpus luteum is related to luteal function separately from the function of vitamin A. These observational studies support the hypothesis that β-carotene contributes to luteal activity and cow reproduction by enhancing the process of sex steroid hormone synthesis in the corpus luteum due to its antioxidant properties.

## 5. Relevance of β-Carotene to Ovarian Activity

The effect of β-carotene on ovarian activity has also been studied at the organ level in cattle. Studies on the role of β-carotene in ovarian follicles in cattle have not revealed any relationship between the β-carotene concentration in follicular fluid and follicle size; however, the vitamin A concentration in the follicular fluid was high in larger follicles [26]. Cleave of β-carotene to vitamin A was efficient in the follicular fluid of larger preovulatory follicles [27]. The concentration of vitamin A in follicular fluid is related to the degree of atresia of the ovarian follicle [28]. Plasma vitamin A levels were high in proestrus and estrus when follicular activity dominated [25]. Thus, these studies support the possibility that β-carotene is locally converted to vitamin A in the follicle, which may affect follicle recruitment, selection, and growth. Therefore, β-carotene availability is potentially related to follicle dynamics in cows. The effects of vitamin A on oocyte cytoplasmic maturation have been reviewed by Ikeda et al. (2005) [29]. Based on the results of these studies, β-carotene provides follicles with moderate amounts of vitamin A and supports cyclic ovarian activity.

Wang et al. (1982) [30] reported the effects of β-carotene supplementation on estrus-induced treatments by prostaglandin F2α (PGF2α; PG) injection. They found that β-carotene supplementation extended the interval between PG injection and estrus, between PG injection and the LH peak, and between PG injection and ovulation while decreasing the interval between estrus and ovulation. The estrus intensity was lower, and the interval between the LH peak and ovulation was longer in herds without β-carotene supplementation than in herds with β-carotene supplementation from 5 to 15 months of age [7]. Although other studies have not shown that β-carotene supplementation affects ovarian activity [10,12,31,32], β-carotene may increase luteal development and P4 production by increasing steroidogenesis through its antioxidant effects, which increases the time required for luteolysis. On the other hand, after luteolysis, β-carotene possibly assists in rapid dominant follicle development and increased estrogen production, presumably increasing estrus intensity and causing an earlier LH surge and ovulation due to a positive estrogen feedback system.

## 6. Recent Progress in Understanding the Mechanism of β-Carotene in Cattle Reproduction

β-carotene and vitamin A concentrations in blood decrease during the peripartum period [33,34,35,36]. Some researchers have suggested the involvement of β-carotene in the recovery of ovarian activity and uterine involution postpartum. The number of days to third ovulation and number of days to conception from parturition were higher in herds with lower serum β-carotene concentrations than in herds whose serum concentrations were higher [37]. Kawashima et al. (2009) [38] reported that postpartum anovulatory cows in the first follicular wave had lower plasma β-carotene concentrations in the prepartum than the ovulatory cows. They also reported that cows fed 2000 mg/day of synthetic β-carotene for three weeks before calving showed apparent luteal activity within three weeks postpartum compared with cows without β-carotene supplementation [39]. Kaewlamun et al. (2011) [40] did not observe an effect of feeding β-carotene before calving on ovarian activity, P_4_ production, or early first ovulation postpartum; however, they found that β-carotene supplementation positively affected blood hydroxyproline, which is an indicator of uterine involution and the percentage of polymorphonuclear leucocytes in both the uterus and cervix. Previously, Rakes et al. (1985) [41] reported that β-carotene supplementation was beneficial for cervical recovery postpartum. Associations of the early onset of postpartum luteal activity with dietary intake or plasma concentrations of β-carotene, vitamin A, vitamin E, and with plasma antioxidant capacity have also been reported in dairy cattle [42].

β-carotene may function to prevent periparturient disease. Previous studies suggested that consuming β-carotene reduces the incidence or severity of mastitis [15,43], while other studies did not find any effect of β-carotene on the incidence of periparturient disease [35,44,45]. Dairy cows fed 300 or 500 mg/day of synthetic β-carotene from 21 days prepartum to 70 days postpartum had a significantly reduced incidence of retained placenta and mastitis and a lower number of days to first estrus after parturition [46]. Prepartum feeding of β-carotene to cows reduced the incidence of placenta retention in multiparous cows, although β-carotene did not affect reproductive performance [47]. In addition, β-carotene supplementation improved luteal development, P_4_ production, and the conception rate in long-term repeat-bred cows with low blood β-carotene concentrations. This effect was enhanced when vitamin A and β-carotene were fed together [48]. Administration of β-carotene effectively treats follicular cysts and improves subsequent conception rates [49].

β-carotene may also play an essential role in establishing and maintaining pregnancy. β-carotene injection in the rump with induction of the second corpus luteum by GnRH injection after artificial insemination increased luteal size and luteal blood flow [50]. Similarly, β-carotene supplementation resulted in health and nutritional improvements, such as lower somatic cell counts in milk, lower serum beta-hydroxybutyric acid levels, earlier first estrus postpartum, and higher P_4_ levels in milk on the 23rd day after insemination, which favor the maintenance of pregnancy [51]. Cows with higher plasma β-carotene concentrations at the time of artificial insemination had higher rates of pregnancy, lower rates of early pregnancy loss, and higher plasma pregnancy-related glycoprotein concentrations than cows with lower plasma β-carotene concentrations [52]. Thus, β-carotene not only assists directly in the production of sex steroid hormones at the cellular level but is also indirectly involved in a series of reproductive processes, such as reducing the incidence of periparturient disease, recovering basal reproductive function postpartum, subsequently achieving fertility success, and establishing or maintaining pregnancy.

Based on these scientific findings, field studies using large commercial herds [53,54,55] or at regional scales using local breeds [56,57,58] have been conducted to determine the effect of β-carotene on cow reproduction in recent years. The increase in these types of studies may reflect the development and widespread use of β-carotene supplementation at commercial scales. An advantage of using larger cattle herds is that the effectiveness of β-carotene for reproduction can be examined with less influence from individual variability and short-term environmental fluctuations. Studies at the regional scale, including studies on local breeds, feeds, and feeding management, could also promote field applications of β-carotene supplementation. Although many of these studies have supported the function of β-carotene in cattle reproduction, some studies have not yet found any association between β-carotene and reproduction [59,60]). This inconsistency implies a potential prerequisite factor that is needed for β-carotene to function as an active substance in the reproductive performance of cows.

The rates of conception or pregnancy in cattle fed β-carotene supplements reported in previous studies are summarized in Table 1. The results in the table show that β-carotene is associated with cattle reproduction. Although many reports have questioned such an association, some of them also showed numerically higher conception rates in cattle fed β-carotene. This conflicting result among studies may be caused by differences in the amount and duration of β-carotene supplementation, basal β-carotene levels in the dairy or beef cow, reproductive management, and basal conception rate on farms. Thus, these preconditions should be considered when evaluating the efficacy of β-carotene for cattle reproduction.

## 7. Possible Factors Affecting the Effectiveness of β-Carotene in Cattle Reproduction

### 7.1. Basal β-Carotene Intake and Vitamin A Status

As noted above, basal β-carotene intake and vitamin A status in the body are the main factors that make the effect of β-carotene supplementation on cattle reproduction unclear. Controlling dietary β-carotene levels in basal feeds is difficult. The main feed sources of β-carotene are forages, including fresh grass, silage, and hay, while concentrates contain less β-carotene due to degradation during heat processing [61]. Forages such as hay and silage often lose β-carotene during the wilting, storage, and feeding processes [62]. Since silage requires less wilting time than hay, silage has a higher β-carotene content than hay, and different wilting periods result in variations in β-carotene content in silages [37]. Moreover, imported hay has a lower β-carotene content than locally produced hay [63]. This is probably because imported hay is exposed to heat and air longer than locally produced hay during transportation and storage.

The intake of β-carotene reflects blood levels of β-carotene [37,62]. According to an investigation of 2467 dairy cows from 127 farms in Belgium, Germany, Italy, and the Netherlands [64], approximately 40% of cows are β-carotene deficient (i.e., blood concentration below 3.5 mg/L), and this percentage varies greatly with country and feeding method. Farm-specific differences in blood β-carotene concentrations have also been reported in Japan [65], Slovenia [66], and South Africa [67]. Interestingly, blood β-carotene levels depend directly on the type of roughage. They also indirectly depend on the natural, geographical, and historical background of roughage utilization, such as pasture utilization and traditional feeding methods, to adapt to different climates. Therefore, the β-carotene content in basal forages and the resulting basal blood β-carotene levels are the main factors that make evaluating the effectiveness of β-carotene supplementation on cattle reproduction difficult. Thus, real-time monitoring of the β-carotene content in forages and animal blood is essential for determining the effect of β-carotene on cattle reproduction. Currently, portable, simple measuring devices have been developed for clinical use [68] and are also used in many academic studies.

The variation in the conversion efficiency of β-carotene to vitamin A is another factor that makes the function of β-carotene in reproduction unclear. There has long been concern that the efficacy of β-carotene depends on whether the level of vitamin A is sufficient in the cow’s body [9,69]. The importance of vitamin A for animal health has been recognized in recent years, and most commercial formula feeds contain sufficient synthetic vitamin A. However, the effectiveness of β-carotene supplementation on vitamin A sufficiency remains to be explained. An observational study by Strickland et al. (2020) [70] reported no difference in serum vitamin A concentrations between farms despite the differences in formulated vitamin A supplementation. This is probably due to the intervention of a feedback system that regulates the vitamin A metabolism.

In mammals, β-carotene oxygenase 1 (BCO1) and β-carotene oxygenase 2 (BCO2) are the primary enzymes that metabolize β-carotene [71,72]. In mice, there is a negative feedback mechanism that regulates β-carotene absorption and metabolism. Retinoic acid, which is the final metabolite of vitamin A, suppresses the expression of BCO1 and scavenger receptor class B type 1 (SRBI), which facilitates the absorption of β-carotene via the transcriptional regulator ISX [73]. Cattle also contain BCO1, BCO2, and SRBI [74], and the regulatory genes of these enzymes are associated with β-carotene content and its relevant color in milk and fat [74,75]. Additionally, β-carotene supplementation increases BCO1 gene expression in beef cattle [76]. A review by Shastak and Pelletier [5] also explained how BCO1 regulates the absorption and metabolic mechanisms of β-carotene in vertebrates, including cattle.

Although further in vivo studies are needed to validate the existence of this regulatory system, β-carotene and vitamins likely function via a similar mechanism in cattle bodies. When vitamin A meets the required level, β-carotene absorption in the gut and metabolism of vitamin A may be inhibited. In other words, if vitamin A levels are below the required level, β-carotene is likely to function more as a source of vitamin A. Thus, when considering β-carotene supplementation for cattle reproduction, monitoring the levels of both β-carotene and vitamin A in feed and cattle blood should be recommended for farms.

### 7.2. Lactation, Age and Parity

The lactation status, age, and parity of animals are other factors that influence the actions of β-carotene in cattle reproductive performance. Plasma β-carotene and vitamin A concentrations are influenced by milk yield, lactation days, and animal age [9]. These external factors may be related mainly to oxidative stress. As an antioxidant, β-carotene protects normal physiological mechanisms from oxidative stress caused by reactive oxygen species and maintains the health of cattle [77]. In repeat breeders who fail to conceive and return to estrus despite clinical normality, the levels of free radicals have been reported to be high [78]. Similarly, in high-yielding milk cows, the conception rate is affected by heat stress, parity, the number of inseminations, and perinatal disease [79]. High-yielding milk cows produce substantial amounts of free radicals and oxidative stress during milk production, which leads to a decrease in reproductive function [80]. β-carotene and vitamin A are present in milk, and their transfer rate from plasma to milk varies throughout the lactation stage [81]. Milk consumes β-carotene as a component in milk through increased oxidative stress. In addition, dietary-induced negative energy balance in nonlactating dairy cattle decreases β-carotene concentrations in follicular fluid and increases systemic oxidative stress levels, but β-carotene supplementation substantially improves the availability of β-carotene and retinol in the oocyte microenvironment in nonlactating dairy cows [82].

Age also affects the serum concentrations of β-carotene and vitamin A in cattle, presumably related to antioxidant consumption due to age-related oxidation in the body [83]. Lower serum vitamin A concentrations have been reported in multiparous cows that had produced three or more offspring than in primiparous cows or multiparous cows that had produced two offspring, implying an increase in the transfer rate of vitamin A to colostrum with age [72]. Thus, age and parity may increase oxidative stress due to increased milk production or aging, which may lead to a deficiency of β-carotene in the body and reduced reproductive efficiency in cattle.

### 7.3. Seasons

The relationship between β-carotene and reproduction may be influenced by season. The most prominent season is summer [84], when feeding β-carotene improves reproductive performance [85]. In winter, luteal function decreases due to low β-carotene levels, possibly caused by less β-carotene intake [86]. Oxidative stress can be an external factor underlying seasonal effects. In general, seasonal oxidative stress causes reduced reproductive performance [87], which is improved by antioxidant vitamins [88]. In other words, the effectiveness of β-carotene on cattle reproduction may be evident by the increase in oxidative stress in the body due to summer heat stress. On the other hand, blood [89] and luteal [90] concentrations of β-carotene are higher in warmer months because fresh grasses that are rich in β-carotene may grow well in these months. However, the effect of season with mature grasses on the β-carotene content of forage is still controversial [62]. Therefore, the seasonal effects on the relationship between β-carotene and reproduction may depend on the balance between the consumption of β-carotene due to heat stress and the supply of β-carotene from basal forage.

## 8. Conclusions

**Figure 2:**
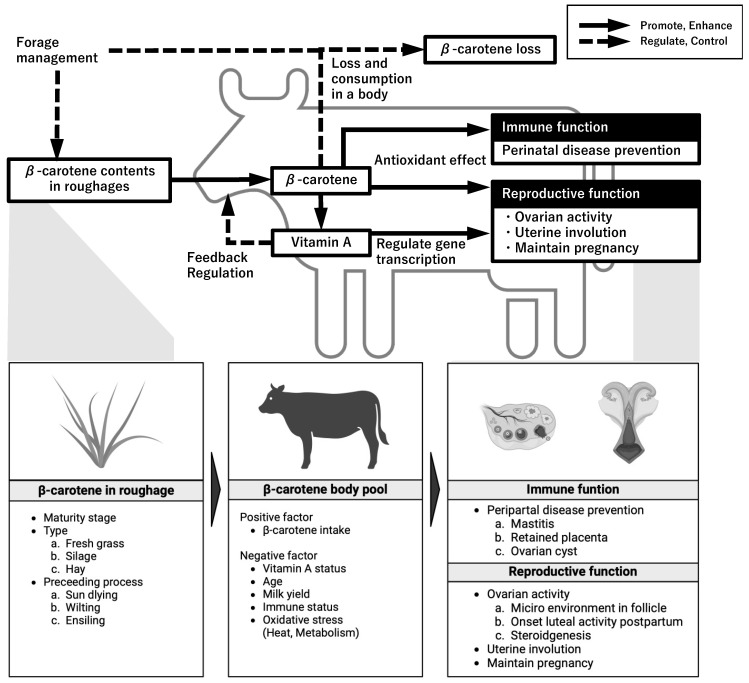
Relationships between β-carotene and cow reproduction. Solid arrows indicate promotion or enhancement. Dotted arrows indicate regulation or control. Created with BioRender.com.

The relationships between β-carotene and cow reproduction are shown in Figure 2. At the molecular level, β-carotene supports reproductive function, especially luteal function, in cattle by protecting synthetic enzymes related to sex steroid hormone production from reactive oxygen species through its antioxidant properties. At the individual level, β-carotene supports the ovarian cycle in cows by suppressing oxidative stress and enhancing immune function. This effect of β-carotene is particularly pronounced during the perinatal period. Future research on the effects of β-carotene on ovarian and uterine function during conception and pregnancy maintenance is promising. The effects of β-carotene on reproductive performance have not always been substantiated on farms. They appear to be influenced by the physiological state of the cow, such as lactation and age, and the feeding management on the farm, including the β-carotene content in roughage and the vitamin A sufficiency level in the body. In the future, the effects of β-carotene supplementation on reproductive performance from multiple perspectives, such as β-carotene sufficiency and the balance between β-carotene intake from roughage and β-carotene consumption in the body, need to be clarified.

## Figures and Tables

**Table 1:** The effect of β-carotene supplementation on conception rate in previous studies.

Study	Cattle Type	Age	β-Carotene Group				Non-β-Carotene Group				Significant Differences in Conception Rate (*p*-Value)
			n ^1^	β-Carotene Supplementation (mg/day)	Blood β-Carotene conc. (μg/dL)	Conception Rate (%)	n	Vitamin A Supplementation as a Control (IU/day) ^2^	Blood β-Carotene conc. (μg/dL)	Conception Rate (%)	
Folman et al. (1979) [10]	Dairy Heifer	7–17 mo ^3^	7	105 ^4^	63–219	78 (+9) ^5^	11	40,800 ^4^	2–6	69	N.S. ^6^
Wang et al. (1982) [30]	Dairy Heifer	12–19 mo	9	300	301	44 (+0)	9	-	228	44	N.S.
		14–19 mo	7		192	71 (+14)	7	-	132	57	N.S.
		12–16 mo	7		194	75 (+8)	9	-	90	67	N.S.
Ducker et al. (1984) [13]	Dairy Heifer	19–24 mo	40	300	273–928	53 (+8)	40	60,000	142–430	45	N.S.
		12–14 mo	40		330-909	58 (+8)	40	60,000	58–364	50	N.S.
Ascarelli et al. (1985) [8]	Dairy Cow	Multiparious	27	500 (dry cow) 750 (lactating cow)	286–493	59 (+18)/1st AI 55 (+17)/All AI	27	200,000 (dry cow) 300,000 (lactating cow)	22–46	41/1st AI 38/All AI	N.S./1st AI *p* < 0.05/All AI
			28	500 (dry cow) 750 (lactating cow)	341-546	36 (+3)/1st AI 43 (+6)/All AI	28	200,000 (dry cow) 300,000 (lactating cow)	18–34	33/1st AI 37/All AI	N.S.
Akordor et al. (1986) [44]	Dairy Cow	32–87 mo	28	400	191	54 (+0)/1st AI 79 (+12)/1-2nd AI 93 (+22)/1-3rd AI	24	160,000	62	54/1st AI 67/1-2nd AI 71/1-3rd AI	N.S./1st AI N.S./1–2nd AI *p* < 0.05/1–3rd AI
Folman et al. (1987) [11]	Dairy Cow	Multiparious	55	500 (before calving) 700 (after calving)	393–657	22 (−24)/1st AI 36 (−33)/2nd AI 28 (−26)/All AI	50	200,581 (before calving) 279,069 (after calving)	78–107	46/1st AI 69/2nd AI 54/All AI	*p* = 0.025/1st AI *p* = 0.025/2nd AI *p* = 0.001/All AI
Wang et al. (1988) [32]	Dairy Heifer	13–18 mo	9	300	127	67 (+11)	9	-	86	56	N.S.
		14–16 mo	10	300	158	67 (+45)	10	-	127	22	
		11–15 mo	9	600	173	44 (+0)	9	-	46	44	
Bian et al. (2007) [46]	Dairy Cow	Multiparious	38 ^7^	300	-	36 (+12)	38 ^7^	-	-	24	*p* < 0.05
De Ondarza et al. (2009) [53]	Dairy Cow	Multiparious	421 ^8^	425	275–356	21 ^9^ (+1)	419 ^8^	-	171–247	20 ^9^	N.S.
Oliveira et al. (2015) [47]	Dairy Heifer	Primiparious	28	1200	202–238	43 (−7)	30	-	155–226	50	N.S.
		Multiparious	74	1200	208–254	34 (+7)	84	-	171–229	27	N.S.
Lee et al. (2021) [57]	Beef	Multiparious	100	400	429	34 (+9)	99	-	103	25	*p* = 0.024
Bhateshwar et al. (2021) [56]	Dairy	Lactating	12	500	287-389	58 (+25)/1st AI 25 (+8)/2nd AI 83 (+33)/All AI	12	-	178-264	33/1st AI 17/2nd AI 50/All AI	N.S./1st AI N.S./2nd AI *p* < 0.001/All AI
Khemarach et al. (2021) [55]	Dairy Cow	-	200	400	-	90 ^9^ (+15)	200	-	-	75 ^9^	*p* < 0.01
Prom et al. (2022) [60]	Dairy Cow	Multiparious	47	800	170	68 ^9,10^ (−13)	47	-	59	81 ^9,10^	N.S.

^1^—number of animals; ^2^—1 IU = 0.3 μg of retinol, 1 IU = 0.344 μg of retinyl acetate [2]; ^3^—months; ^4^—the value was calculated from the amount supplementation per body weight and the body weight at first insemination; ^5^—values in parentheses indicate the difference from non-β-carotene group; ^6^—not significant (*p* > 0.05); ^7^—the text was marked “76 multiparous cows were randomly divided into 2 groups”; ^8^—pregnant eligible; ^9^—pregnancy rate; ^10^—calculated from the number of pregnant, bred and not pregnant, and not bred.

## Data Availability

No new data were created or analyzed in this study.
